# Artificial neuromuscular fibers by multilayered coaxial integration with dynamic adaption

**DOI:** 10.1126/sciadv.abq7703

**Published:** 2022-11-16

**Authors:** Lizhong Dong, Ming Ren, Yulian Wang, Guanghua Wang, Shiqin Zhang, Xulin Wei, Jianfeng He, Bo Cui, Yueran Zhao, Panpan Xu, Xiaona Wang, Jiangtao Di, Qingwen Li

**Affiliations:** ^1^ School of Nano-Technology and Nano-Bionics, University of Science and Technology of China, Hefei 230026, China.; ^2^ Advanced Materials Division, Key Laboratory of Multifunctional Nanomaterials and Smart Systems, Suzhou Institute of Nano-Tech and Nano-Bionics, Chinese Academy of Sciences, Suzhou 215123, China.; ^3^ Division of Nanomaterials and Jiangxi Key Lab of Carbonene Materials, Jiangxi Institute of Nanotechnology, Nanchang 330200, China.

## Abstract

Integrating sense in a thin artificial muscle fiber for environmental adaption and actuation path tracing, as a snail tentacle does, is highly needed but still challenging because of the interfacing mismatch between the fiber’s actuation and sensing components. Here, we report an artificial neuromuscular fiber by wrapping a carbon nanotube (CNT) fiber core in sequence with an elastomer layer, a nanofiber network, and an MXene/CNT thin sheath, achieving the ingenious sense-judge-act intelligent system in an elastic fiber. The CNT/elastomer components provide actuation, and the sheath enables touch/stretch perception and hysteresis-free cyclic actuation tracing due to its strain-dependent resistance. As a whole, the coaxial structure builds a dielectric capacitor that enables sensitive touchless perception. The key to seamless integration is to use a nanofiber interface that allows the sensing layer to adaptively trace but not restrict actuation. This work provides promising solutions for closed-loop control for future intelligent soft robots.

## Teaser

An artificial neuromuscular fiber by a coaxial structure integrates perception-action-feedback function on the same fiber.

## INTRODUCTION

Living animals have a neuromuscular system that senses external changes and executes actions through the synergy of nerves and muscles (*1*–*3*). For example, the snail tentacles shrink when touched or are very closely approached. This irritability helps the snail avoid sudden dangers and increases its adaptability to environmental changes. Considering the fast development of soft robots, such a simple fusion system is highly needed to make robotics more intelligent, advanced, and realistic (*1*, *4*, *5*). While classic rigid robots can achieve the perception-actuation-feedback function by motors with the drivetrain, force sensors, and camera systems (*6*–*8*), the integration of such components often limits the miniaturization and flexibility of these robots. Alternatively, artificial muscle fibers have opened up promising opportunities for preparing more compact and flexible drive units (*9*–*15*). Like natural muscles, artificial muscle fibers provide large and reversible contractile strokes while lifting heavy loads when driven by the stimuli such as heat (*9*, *15*–*17*), humidity (*18*–*20*), solvent (*17*, *21*–*23*), and double-layer charging (*10*, *17*, *24*). For example, Haines *et al.* (*9*) demonstrated that electrothermal nylon 6,6 artificial muscle fibers could achieve a maximum specific work of 2.48 J g^−1^ and a maximum mechanical output power of 27.1 W g^−1^. By designing biomimetic carbon nanotube (CNT) yarns, Son *et al.* (*20*) reported a rapidly electrothermally recoverable high-power hydro-actuator that demonstrated a full-time power density of 143.8 W kg^−1^ and a high actuation frequency of 0.17 Hz. Cui *et al.* (*23*) prepared tendril-like hydrogel artificial muscles in response to ethanol and water with large stroke (87%) and shape memory property. Recently, the electrochemical CNT yarn muscles with a layered inner structure reported by our group achieved a 62.4% ultralarge contraction (*25*).

Despite the well-developed actuation performance, the functional integration of sense in an artificial muscle fiber for environmental change adaption and actuation path tracing is still of great challenge and has not been reported. Very recently, a few multifunctional film actuators were prepared by the combination of independent pressure sensing units and actuating units. For example, He *et al.* (*3*) mimicked the somatic reflex arc to prepare an electrochemical actuator in response to the stimulation of tactile pressure. This device consisted of three independent electronic components: a pressure sensing unit, a threshold controlling unit, and an electrochemical actuating unit. When the pressure sensor detected a pressure higher than the stimulation threshold, the threshold controlling unit could be activated; subsequently, the actuator was triggered to complete the motion. Zhou *et al.* (*26*) imitated the mimosa to propose a pressure-perceptive actuator driven electrothermally. The artful combination of actuating and sensing units permitted the deformation of the actuator to be regulated by a pressure stimulus. For the functional integration in a fiber-shaped artificial muscle, the main obstacle is the interfacing mismatch between the actuation and sensing components in a thin fiber. Contractile artificial muscle fibers commonly use a coiled structure that converts the radial expansion through untwisting into axial contractile strokes (*27*). The complex structural deformation during actuation makes it impossible to simply attach sensing layers on a muscle fiber as that done for the film actuators. Because of the poor actuation-sensing interface, the self-sensing function of previously reported artificial muscle fibers easily failed during cycling and the environmental sensation was not available (*16*, *28*, *29*). Therefore, the sensing layers should be dynamically adapted to the structural evolution of artificial muscle fibers during the actuation by a well-bridged interface.

To overcome these challenges, we designed an artificial neuromuscular fiber through a nanofiber-interfaced triple-layered coaxial structure. An adaptive polyacrylonitrile (PAN) nanofiber layer was used to dynamically bridge the polydimethylsiloxane (PDMS) actuation layer and the MXene/CNT sensing layer, which greatly increases the stability of artificial neuromuscular fibers and the matching of real-time path tracing during actuating. The artificial neuromuscular fiber can perceive multi-somatosensory excitation signals (proximity, stretch, and pressure) to execute electrothermally driven actuation while providing a fully linear real-time path tracing of the whole actuating process. In particular, the introduction of the proximity-perceiving mode enables artificial neuromuscular fibers to make a command to actuate or not by recognizing the speed of approaching objects. We believe that this all-in-one artificial neuromuscular fiber can reduce the complexity of the sensing and actuating units of intelligent structures and systems, thereby facilitating the development of next-generation intelligent robots.

## RESULTS

### Structural design and characterization

**Fig. 1.**
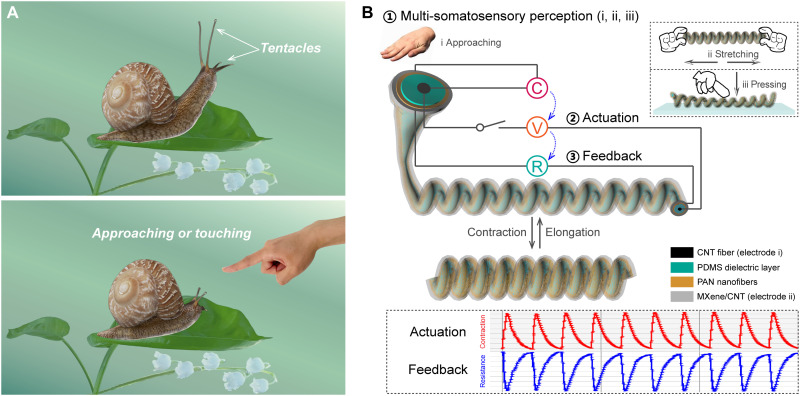
Schematic showing the concept of the artificial neuromuscular fiber with integrated perception-actuation-feedback function. (**A**) Perception and contraction of snail tentacles. (**B**) Schematic illustration of the designed structure and the working mechanism of the artificial neuromuscular fiber that integrates perception-actuation-feedback function.

Figure 1A shows the response of a snail when its tentacles touch an external object. We mimic this interesting and important natural behavior using an artificial muscle fiber that has a triple-layered coaxial structure (Fig. 1B). The components of the coaxial structure of the fiber from the inside to the outside are a CNT core, an inner PDMS layer, a three-dimensional (3D) PAN nanofiber interface layer, and an MXene/CNT outer sheath. The PDMS layer Joule-heated by the CNT fiber core provides volume change–induced actuation. The MXene/CNT outer sheath functions as the touch/stretch perception and real-time path tracing during actuation since its resistance is strain dependent. The coaxial structure (CNT electrode, PDMS dielectric layer, and MXene/CNT electrode) can work as a dielectric capacitor for sensitive touchless perception. Through this highly integrated ingenious design, an all-in-one artificial neuromuscular fiber is achieved, which can perceive multi-somatosensory excitation signals (proximity, stretch, and pressure) to execute actuation commands while providing a fully linear real-time path tracing of the whole actuating process.

**Fig. 2.**
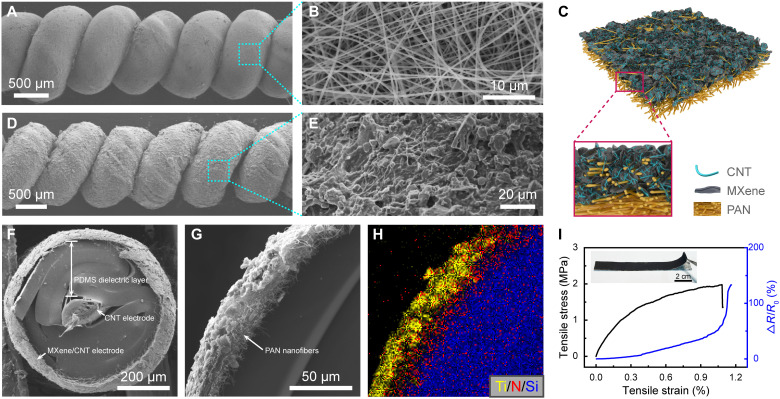
Preparation and characterization of the artificial neuromuscular fiber. (**A**) SEM image of the PAN-wrapped fiber. (**B**) SEM image of the PAN layer showing a 3D porous structure. (**C**) Schematic illustration of the surface structure of the artificial neuromuscular fiber. (**D**) SEM image of artificial neuromuscular fiber. (**E**) SEM image of the MXene/CNT conductive layer. (**F**) Cross-sectional SEM images of the whole and (**G**) the edge of artificial neuromuscular fiber. (**H**) Elemental mapping (blue, red, and yellow spots correspond to Ti, N, and Si, respectively) over the area in (G). (**I**) Mechanical properties and the change of relative resistance as a function of strain for an MXene/CNT-coated self-supporting PAN film.

The preparation of the artificial neuromuscular fiber is illustrated in fig. S1 (see Materials and Methods for details). Briefly, after wrapping a CNT fiber with a PDMS thin ribbon, a layer of PAN nanofibers was deposited on the outer surface of the fiber by electrospinning. Then, the composite fiber was coiled by twisting under tension. The MXene/CNT conductive outer sheath was coated on the coiled fiber by dipping the fiber in the MXene/CNT solution, followed by drying it in the air. Electrospun PAN nanofibers formed a 3D network structure (fig. S2), and the orientation of PAN nanofibers had not changed much and still maintained a porous network structure after fiber coiling (Fig. 2, A and B). This interwoven network structure could help to form a continuous MXene/CNT conductive layer on the surface of the artificial neuromuscular fiber since the microscale pores formed by the nanofibers could grasp the MXene/CNT composites (Fig. 2C). Figure 2 (D and E) confirms that the conductive network formed by the MXene/CNT was uniformly attached to the surface of the nanofiber network layer. This is of great importance for the MXene/CNT sheath to work as a sensing layer and an electrode for the dielectric capacitor. The utilization of the MXene/CNT sheath as the sensing layer is due to the 3D conductive structures composed of multilayer structures, and CNT can respond to very subtle mechanical stimuli. The multilayer structure of MXene was evenly filled with CNTs to further increase the sensitivity of the strain-dependent resistance (the inset of fig. S3). Raman spectroscopy in fig. S3 showed that typical peaks at 154 and 630 cm^−1^ were assigned to the Ti-C and C-C vibrations of the oxygen-terminated MXene, respectively (*30*, *31*). The peaks at 398 and 510 cm^−1^ were attributed to the vibrations of O atoms, and the peak at 180 cm^−1^ reflected the radial breathing mode of CNT (*30*, *32*).

The cross section of the artificial neuromuscular fiber shows the designed triple-layered coaxial structure, including the CNT fiber core, the relatively thick PDMS actuation layer, the PAN nanofiber network interface layer, and the MXene/CNT sensing sheath (Fig. 2F). The thickness of the nanofiber-supported MXene/CNT sheath was ~25 μm, which was only ^1^/_10_ of the actuation layer. The high-resolution scanning electron microscopy (SEM) image (Fig. 2G) and the corresponding elemental distribution analysis (Fig. 2H) confirmed that the MXene/CNT composite flakes were embedded in the network of the PAN nanofibers. Figure 2I shows the stress and resistance change as a function of tensile strain for a thin nanofiber-supported MXene/CNT film. The resistance linearly and smoothly increased with the increase of tensile strain. This indicates that the applied tensile stress was efficiently transported through the whole film and thus caused steady structural change for resistance sensing. The composite film also had a very low modulus of ~460 MPa, which could show very little restriction on actuation especially when the film was thin.

### Multimode sense of press, stretch, and proximity

**Fig. 3.**
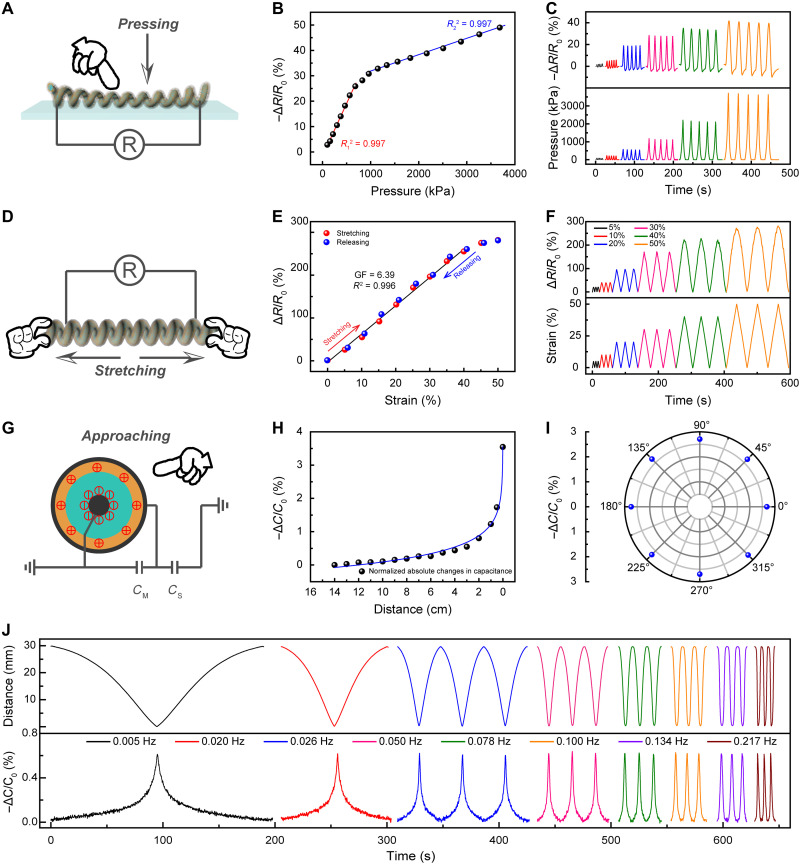
Pressure- and strain-perceptive properties in the touch mode; proximity-perceptive properties in the touchless mode of the artificial neuromuscular fiber. (**A**) Schematic illustration of the working mechanism for the pressure perception. (**B**) Ultrahigh linear sensitivity of the pressure perception. (**C**) Change of relative resistance as a function of different pressures for the artificial neuromuscular fiber. (**D**) Schematic illustration of the working mechanism for the strain perception. (**E**) Ultrahigh linear sensitivity of the artificial neuromuscular fiber under strain from 0 to 40%. (**F**) Response of the relative resistance at strains of 5, 10, 20, 30, 40, and 50%. (**G**) Working mechanism of the proximity perception and equivalent circuit of capacitance for the measurement of proximity. (**H**) Change of relative capacitance with respect to the distance of approaching hand. (**I**) Change of relative capacitance with respect to the same distance of approaching hand at different angles. (**J**) Relationship between the sensitivity and the approaching speed of the perception signal.

The piezoresistance properties of the coaxial artificial neuromuscular fiber by press and stretch were first investigated. The fiber was pressed with increasing pressure (here, the pressure was calculated by dividing the applied force by the apparent cross-sectional area of the coiled fiber, as schematically illustrated in fig. S4), and the corresponding resistance change was recorded (Fig. 3A). Figure 3B shows the resistance change versus pressure. The resistance increased linearly with the increase of pressure in both the low-pressure region (0 to 962 kPa) and the high-pressure region (962 to 3685 kPa) but with different slopes. This dependence profile was very repeatable during cyclic tests. These results can be attributed to the structural changes in different layers of the artificial neuromuscular fiber. The linear sensitivity in the low-pressure region (coefficient of determination, *R*^2^ = 0.997) could be influenced by the changes in the microstructure of the MXene/CNT layer, while the linear sensitivity in the high-pressure region (*R*^2^ = 0.997) could be mainly derived from the combined effect of structural changes in the PAN nanofibers and PDMS substrates due to the increased pressure. The corresponding real-time resistance change under different pressures of 99, 217, 574, 1141, 2166, and 3718 kPa showed very fast resistance response rates under these wide pressure ranges (Fig. 3C). In addition, the artificial neuromuscular fiber presented a very reliable resistance change at the same pressure (~2100 kPa) but with different press frequencies ranging from 0.043 to 0.46 Hz (fig. S5). Furthermore, 1000 cycles of compression-release tests were performed under a pressure of ~1820 kPa (fig. S6), which indicated good long-term stability.

Figure 3D schematically shows the strain perception of the artificial neuromuscular fiber. The relative resistance change (Δ*R*/*R*_0_, Δ*R = R* − *R*_0_, *R* and *R*_0_ are the real-time resistance and the initial resistance of the sheath, respectively) of the artificial neuromuscular fiber had an excellent linear relationship in the strain range from 0 to 40% (*R*^2^ = 0.996), and the gauge factor [GF = Δ*R*/(ε*R*_0_); ε is the stretch strain] was 6.39 (Fig. 3E). During stretch-release cycles, all the resistance change-strain curves coincided and no resistance hysteresis was observed. The artificial neuromuscular fiber exhibited very repeatable resistance change under different tensile strains (10, 20, 30, 40, and 50%; Fig. 3F) at the same stretching speeds and the same tensile strains at different stretching speeds (5, 10, 20, 50, and 100 μm s^−1^; figs. S7 and S8). The results of 1000 stretch-release cycles that were tested at 20% strain and 10 μm s^−1^ stretch speed showed good stability and reliability of the artificial neuromuscular fiber for strain sensation by resistance change (fig. S9). These results indicate that the piezoresistance of the artificial neuromuscular fibers can respond to wide ranges of pressure and strain with linear correlation and good cyclic reliability. Moreover, because of the deformation of the PDMS layer caused by stretching, there was an excellent linear relationship between the relative capacitance change and tensile strain (fig. S10).

The ingenious design of the coaxial structure allows the artificial muscle fiber to work as a dielectric capacitor to perceive an approaching object. Figure 3G presents the circuit diagram of using an artificial neuromuscular fiber as a capacitor for proximity perception. The sandwich structure provides high sensitivity by using the mutual inductance capacitance generated by the PDMS dielectric layer between the orthogonal electrodes (*33*). Proximity detection can be comprehended as an object inserted in the fringe electric field, causing a decrease in the capacitance of the device (*34*–*36*). Two effective capacitors exist in this simple device: a mutual capacitor (*C*_M_) existing between two carbon-based electrodes and a self-capacitor (*C*_S_) existing between the outer sheath of the artificial neuromuscular fiber and the object (the third electrode). When an object is approached, partially shunting and intercepting the electric field to the ground within a perceptible range occurs. This decreases the electric field strength between the two electrode plates of the *C*_M_ since the *C*_S_ and *C*_M_ are connected in series, thereby decreasing the charge stored in the *C*_M_. Theoretically, any approaching object can be detected by using this same mechanism, because the total capacitance value of the capacitors in series will depend on the dielectric constant of the objects in proximity (*33*).

Figure 3H shows the relative capacitance changes in an artificial neuromuscular fiber with the approach of the hand. The change of relative capacitance increased progressively with the approaching of the hand. In particular, the proximity perception signal was more sensitive to distance in the 1- to 2-cm distance range than the 2- to 14-cm range. This is attributed to the fact that the *C*_M_ decreased logarithmically during the approaching of the hand, which corresponded well to the model developed by Garbini (*37*). The relative capacitance changes of the artificial neuromuscular fiber were further measured when the hand approached the fiber from the circumferential directions (Fig. 3I). It shows that the relative capacitance changes were only related to the distance between the object and the fiber but irrelevant to the approaching direction. This can result from the fiber-based geometric structure.

The proximity perception properties were further analyzed in the recognition of a stainless bar that approaches the fiber with the same proximity distance at different approaching speeds. Figure 3J shows the approaching distance and the corresponding relative capacitance change as a function of time. Although the approaching speeds were different, the capacitance changes were the same when the object approached the fiber at the same distance. The curves of the capacitance changes showed excellent symmetry in a full cycle of approaching and leaving the object. This indicates that the capacitive response of the fiber is very fast.

The integration of proximity-perceiving function in artificial muscle fibers may provide a new mode of interaction and control when the neuromuscular fibers are used for intelligent structures and systems. For example, it can be applied to targeted grasping and evasive actions of intelligent robots as well as intelligent systems to make corresponding execution commands according to the approaching speed of objects.

### Electrothermal actuation

The actuation of these artificial neuromuscular fibers was enabled by Joule-heating the PDMS layers by the CNT fiber cores. For an artificial muscle fiber that has a twist-induced coiled structure, stimulus-induced radial expansion can cause untwisting of the fiber, which thus brings the coils close for lengthwise contraction (*9*, *15*, *17*). A proper load is generally applied to stretch the fiber and separate the coils to provide space for contraction. When the load was small (e.g., <8 g in the present case), Joule heating caused fiber elongation but not contraction (figs. S11 and S12), because the coils still firmly contacted with each other and thermal radial expansion of the PDMS layer pushed adjacent coils for elongation. When the load was large enough to separate the coils, the lengthwise contraction of the fiber was enabled. Unless otherwise mentioned, the following sections focus on reversible contractile actuation.

**Fig. 4.**
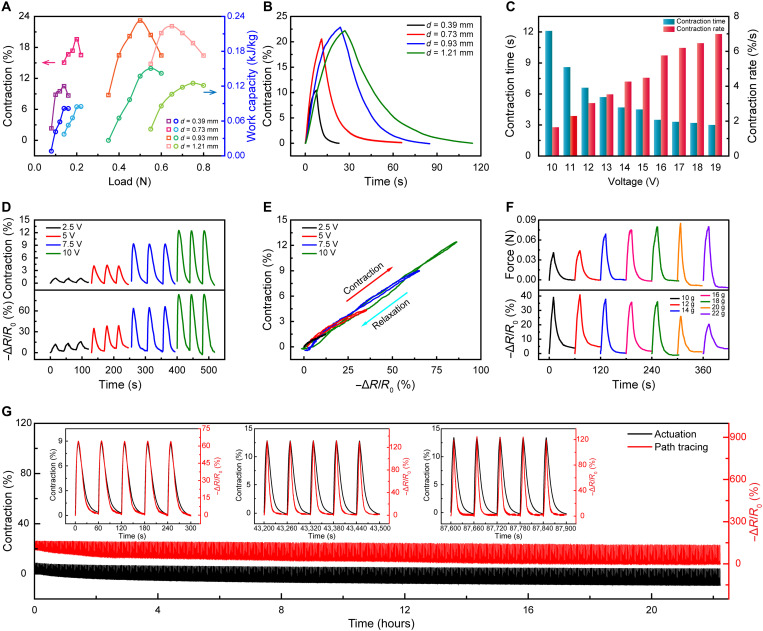
Electrothermal actuation and real-time path tracing properties of the artificial neuromuscular fiber. (**A**) Applied load dependence of contraction (left axis) and corresponding work capacity (right axis) for muscle fibers with different PDMS sheath thicknesses (*d* = 0.39, 0.73, 0.93, and 1.21 mm), while a current of 0.14 A was applied. (**B**) Maximum contraction versus time of muscle fibers with different PDMS sheath thicknesses at 0.14-A current. (**C**) Contraction time (left axis) and contraction rate (right axis) of the muscle fiber achieving the maximum contraction under 20-g load at different voltages. (**D**) Relative resistance and contraction as a function of time under different voltages and 14-g load. (**E**) Linear relationship between actuation and real-time path tracing of the artificial neuromuscular fiber under different voltages. (**F**) Relative resistance and isometric force as a function of time under different loads at 10 V. (**G**) Cyclic tests of the contraction with real-time path tracing signal (under 10-V voltage with 14-g load, powering on for 8 s and powering off for 52 s) on the artificial neuromuscular fiber. The inset is the first five cycles, the middle five cycles, and the last five cycles of the real-time path tracing signal.

The dependences of the contractile stroke, the contraction/recovery rate, and work capacity on the thickness of the PDMS sheath were investigated (Fig. 4, A to C; figs. S13 to S18; and table S1). The maximum contractile stroke of the muscle fibers increased as the sheath thickness increased from 114 to 384 μm and then started to decay. The muscle fiber with a 384-μm-thick PDMS sheath showed a maximum contraction of 23.2% under a 50-N load when driven by passing through 0.14-A current. The maximum work capacity was 0.15 kJ/kg when the applied load was 55 N (Fig. 4A). Under the safe voltage that did not destroy the structure of the muscle fibers, the contraction rate was improved by increasing voltage (figs. S16 and S17). The muscle fibers with a PDMS sheath thickness of 284 μm (*d* = 0.73 mm) rapidly contracted to the maximum contractile stroke of ~20% at 19 V within 3 s, and the corresponding contraction rate was 7%/s (Fig. 4C).

### Actuation-sense integration and application demonstrations

The strain-dependent piezoresistance of the sheath layer can be used to trace the actuation path of the artificial neuromuscular fibers. Before this utilization, the temperature coefficient of resistance of the MXene/CNT sheath should be tuned to be small enough to minimize the influence of temperature-induced resistance change on the total resistance change during thermal actuation. The resistance change in a temperature range used for thermal actuation was thus measured for the sheath layers with different weight ratios between MXene and CNTs (figs. S19 to S23). As shown in fig. S23, the relative resistance change of the sheath layer with MXene:CNT = 5:1 at 150°C was less than 5%. Compared to the relative resistance change (~80%) of path tracing signals, this small temperature-induced resistance change is negligible.

Figure 4D shows the time dependence of the resistance change of the sheath and the actuation path of the artificial neuromuscular fiber that was driven electrothermally by applying different voltages (2.5, 5, 7.5, and 10 V) under 14-g load. The resistance change showed the in-phase trend as the contractile stroke. Notably, Fig. 4E further indicates that the resistance change linearly traced the full actuation path of the artificial neuromuscular fiber, and nearly no hysteresis in a full actuation cycle was observed for these neuromuscular fibers that run at different driving voltages. This hysteresis-free actuation trace was also verified in the previous section on stretch perception (Fig. 3E). The linear dependence of the contractile stroke on the resistance change was also achieved when the artificial neuromuscular fiber worked under different loads of 12, 14, 16, 18, and 20 g (figs. S24 and S25). At the conditions of 15-V driving voltage and 20-g load, the artificial neuromuscular fiber reached the maximum contraction of 20% within 5 s and a hysteresis-free path tracing was also achieved (figs. S26 and S27). The real-time path tracing of the artificial neuromuscular fiber with 14-g load under 10-V voltage for ~1500 cycles is shown in Fig. 4G. The contraction of the artificial neuromuscular fiber gradually stabilized after the initial ~100 cycles of contraction/relaxation training, and the real-time path tracking maintained an excellent linear relationship with the actuation during the entire cycling test (the inset of Fig. 4G). SEM images showed that the MXene/CNT sheath still maintained a very intact surface microstructure after ~1500 actuating cycles (fig. S28).

A hysteresis-free path tracing means that the position of the contractile stroke can be readily identified by the resistance change of the fiber sheath no matter whether the artificial neuromuscular fiber is contracting or recovering. This feature is of great importance because the gesture statuses and actions of a robot that serves the artificial neuromuscular fibers as driving components may be illustrated from the information of the resistance change and the change direction. Although the idea of combining actuation and actuation feedback has been reported previously, the relationships between the actuation and actuation feedback have a very large hysteresis (*16*, *28*, *29*). This results in a huge difficulty in distinguishing the muscle position since one resistance change may correspond to two or more different contractile strokes during an actuation cycle. Since the structural changes of a coiled artificial muscle fiber during actuation are very complex, which may include twisting, expansion, and bending, in situ conformally copying the structure change is, therefore, essential for the sheath as a sensor to trace the actuation. For our artificial neuromuscular fiber, the roles of the 3D porous PAN nanofiber layer are not only to increase the bonding with the MXene/CNT outer sheath layer but also to provide sensitive conformal and real-time response to the deformation generated by the PDMS actuating layer. Without this dynamic nanofiber layer, large hysteresis was observed (*29*).

The artificial neuromuscular fiber can generate isometric force when electrothermally heated with the length of the fiber fixed. The relationship between the resistance change of the outer sheath and the isometric force under various conditions was also investigated (Fig. 4F and figs. S29 to S32). A linear dependence of the resistance change on the change of isometric force but with hysteresis was observed for the artificial neuromuscular fiber that was driven by 10-V voltage under 20-g load (fig. S29), and the cyclic test indicated the good stability of the linear dependence (fig. S30). The hysteresis might be due to the irreversible structure change that was caused by the mechanical friction between adjacent coils. This observation is consistent with that of the press-induced resistance change (Fig. 3C and fig. S5). While the length is fixed, the diameter changed when the artificial neuromuscular fiber was Joule-heated because of the large thermal expansion coefficient of PDMS (figs. S31 and S32). The radial expansion caused by the PDMS sheath can lead to the geometrical change of the MXene/CNT sheath and thus resistance change. Furthermore, the path tracing during elongation of the artificial neuromuscular fiber was linear and stable (fig. S33). In addition to the real-time actuation path tracing by piezoresistance, the relative capacitance changes of the artificial neuromuscular fiber also showed the real-time path tracing of the actuation (fig. S34).

**Fig. 5.**
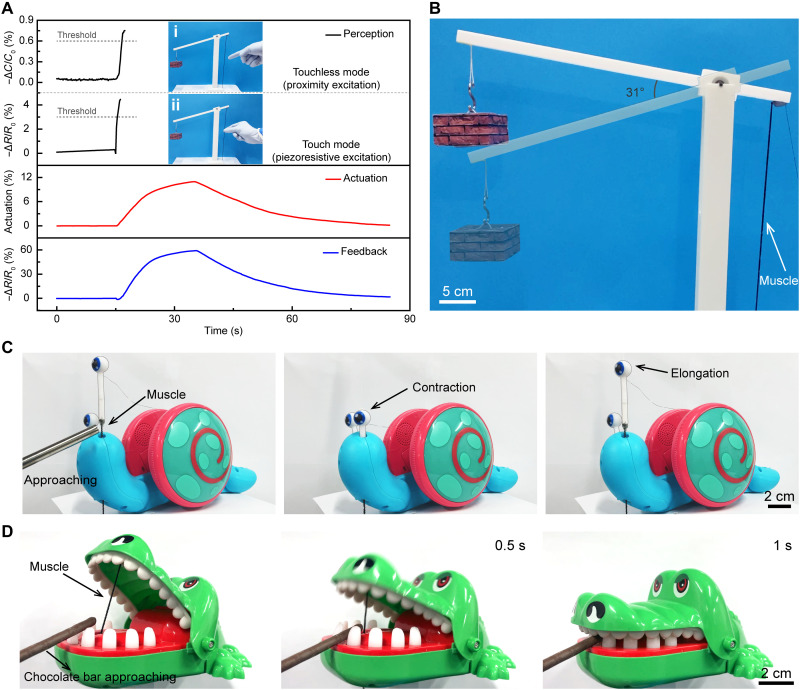
Application demonstrations of the artificial neuromuscular fibers. (**A**) Perception-actuation-feedback signals of a 3D-printed crane working in different modes (touchless mode by perception excitation and touch mode by piezoresistive excitation). (**B**) A crane with perception-actuation-feedback function based on the artificial neuromuscular fiber. (**C**) A snail model with artificial neuromuscular fibers imitating the perception and actuation states of the tentacles of the snail. (**D**) Process of a crocodile mouth capable of preying on the approaching chocolate bar.

To mimic the natural neuromuscular system, the utilization of the multimode sense signals of the press, stretch, and proximity to trigger the actuation of artificial neuromuscular fibers was demonstrated. Figure 5 (A and B) shows an example of the integration of perception, actuation, and feedback functions using a crane model that was driven by an artificial neuromuscular fiber. To facilitate the control of the muscle, we made a control circuit using the STM32F103 development board with a 12-bit analog-to-digital conversion module and the one-way 5-V relay module (fig. S35). When the artificial neuromuscular fiber was subjected to a transient external stimulus either by touch (or approaching), the resistance changes (or capacitance change) could be detected by the fiber. Once the excitation signals reached the set threshold, the artificial neuromuscular fiber was triggered to contract to lift the load by the lever arm (Fig. 5B and movie S1). At the same time, the processes of the load lifting and the fiber actuation were in situ traced by the resistance change of the sensing sheath of the artificial neuromuscular fiber.

The sensitivity of proximity perception signals at different approaching speeds can be used to perceive whether the external action is friendly or dangerous. For example, in our daily life, the handshaking action is a relatively slow process that can be identified by induction signal for low approaching speed (the black curve of Fig. 3J); on the contrary, the hitting action is a relatively fast process that can be identified by induction signal for high approaching speed (the brown curve of Fig. 3J). The ability of artificial neuromuscular fibers to recognize the speed of approaching signals is very important for future smart robots to make a series of actions for environmental adaptation.

We further mimicked the irritability of snail tentacles and the predation behavior of crocodiles using artificial neuromuscular fibers. Artificial neuromuscular fibers were installed on a tentacle of a snail model (Fig. 5C and movie S2). When the tentacle of the snail model perceived a foreign metal rod, the tentacles rapidly contracted with the assistance of artificial neuromuscular fibers to complete the protection of the body. As the stimulation subsides, the tentacle slowly stuck out from the head, corresponding to the muscle recovery process. The predation process of a crocodile model is shown in Fig. 5D and movie S3. When a chocolate bar was fed into the mouth of the crocodile, the crocodile felt the chocolate bar by the capacitance change of the artificial neuromuscular fiber and closed its mouth in 1.5 s, which was enabled by the contraction of the artificial neuromuscular fiber. Hence, the snail model completed the protection of its body against external touch, and the crocodile model completed predation through active perception with the assistance of artificial neuromuscular fibers.

## DISCUSSION

Here, we have successfully demonstrated a concept to more simply and efficiently complete the complex and independent working process of the biological nervous and muscular systems on a smart device. Through the fusion of actuating and sensing units, an artificial neuromuscular fiber with integrated perception-actuation-feedback function on the same fiber is designed, which has the same function as snail tentacles. This artificial neuromuscular fiber can perceive multi-somatosensory excitation signals (proximity, stretch, and pressure) and then execute contractile commands in response to electrothermal stimulation, accompanied by a fully hysteresis-free real-time path tracing of the whole actuation state. The introduction of the 3D adaptive PAN nanofiber interfaces successfully solves the problem of device damage caused by the modulus mismatch between the sensing and actuating materials of current self-sensing artificial muscles. This universal 3D adaptive interface is capable of coupling most of the sensing and actuating functional components together, which preserves the functions of each component for sensory actuation. Compared with conventional perceptive artificial muscles, the ingenious multilayer coaxial design and the synergy of each multifunctional layer provide multimodal perception, especially in proximity perception, and feedback control functions to the artificial neuromuscular fiber. This all-in-one design highlights the lightweight and flexible properties of the artificial neuromuscular fiber, which is expected to replace the traditional bulky and complex actuating and sensing units in intelligent structures and systems, thereby helping advance efforts toward the fields of smart soft robots, human-machine interfaces, and intelligent bionics.

## MATERIALS AND METHODS

### Materials

PAN (molecular weight, 150,000), *N*,*N*-dimethylformamide (DMF), hydrofluoric acid (HF), and sodium dodecylbenzene sulfonate (SDBS) were purchased from Aladdin (Shanghai, China). Ti_3_AlC_2_ (MAX) phase powders, hydroxylated CNTs, and PDMS film were purchased from 11 Technology Co. Ltd. (Jilin, China), XFNANO Materials Technology Co. Ltd. (Nanjing, China), and Baoerde New Material Technology Co. Ltd. (Hangzhou, China), respectively.

### Preparation of CNT fibers and multilayered Ti_3_C_2_T_x_ MXene nanosheets

CNT fibers were prepared by floating catalytic chemical vapor deposition, as we reported previously (*25*). The diameter of the CNT fiber is ~163 μm. Multilayered Ti_3_C_2_T_x_ MXene was synthesized through chemical etching of Ti_3_AlC_2_ (MAX) phase powder with aqueous HF solution, as reported previously (*29*, *38*). The multilayered Ti_3_C_2_T_x_ MXene nanosheets displayed an organ-like structure, which indicated that Al layers had been successfully etched from Ti_3_AlC_2_ (MAX) phase powders, and the interlayer spacing of the MXene powders was completely opened (fig. S36) (*39*). The x-ray diffraction (XRD) pattern of MXene is shown in fig. S37. Compared with the Ti_3_AlC_2_ (MAX) phase, the intensity of the MXene diffraction peaks at 39° decreased significantly, and the (002) diffraction peak shifted toward a smaller angle and was broadened, which also indicated that Al layers had been successfully etched from Ti_3_AlC_2_ (MAX) phase (*40*).

### Fabrication of artificial neuromuscular fibers

Figures S1 and S38 show the preparation of the artificial neuromuscular fibers. A 40-cm-long, 5-mm-wide, and 50-μm-thick PDMS film completely wrapped a 44-cm-long CNT fiber. The entire ribbon was suspended between a clip (10 g) and a stepper motor and then was inserted a twist of 818 turns/m (fig. S38A). Then, 4.8 g of PAN powder was dissolved in 35.2 g of DMF solvent and prepared as a precursor solution with a mass fraction of 12%. The solution was stirred in a water bath at 80°C for 4 hours and then at room temperature for 20 hours. Electrospinning was performed at a flow rate of 0.8 ml hour^−1^ with an applied voltage of 16 kV. The ends of the twisted CNT@PDMS fiber were fixed on a metal fixture and rotated around the axis at 300 rpm to receive electrospinning nanofibers. The twisted CNT@PDMS fiber was kept at a distance of 8 cm from the syringe. The obtained product (fig. S38B) was placed in a vacuum oven and kept at 50°C for 10 hours to remove the residual solvents. Subsequently, the PAN-wrapped fiber was inserted into a twist of 1905 turns/m to form a coiled muscle fiber (fig. S38C). The multilayer MXene powder was added to the CNT dispersion (0.1 weight %, dispersed by SDBS on a 1:1 ratio in water) for uniform ultrasonic dispersion. The PAN-wrapped coiled fiber was coated by this MXene/CNT uniform dispersion to obtain a 3D conductive layer (fig. S38D). As shown in fig. S39, the proportion of each part of the multilayer coiled muscle fiber is 2.98% (CNT), 90.01% (PDMS), 0.69% (PAN), and 6.32% (MXene and CNT), respectively.

### Characterization and measurement

The surface morphology of the prepared muscle fibers was captured by SEM (S-4800, Hitachi). XRD data were recorded by a D8 Advance (Bruker AXS) diffractometer with Cu Kα radiation. The structure characterization was performed by using a Raman spectrometer (HR800 Horiba JY).

The actuating properties were measured by a stress sensor (JZ100, Beijing Xinhang Industrial Electronic Co. Ltd.) and a contactless electromagnetic displacement sensor (M18, Shanghai Muxi Electronic Technology Co. Ltd.). A programmable direct-current (DC) power supply (2280S-60-3, Keithley) was used to provide DC voltage to the artificial neuromuscular fibers. The sensing signals were collected by a digital multimeter (DMM6500, Keithley), a capacitance meter at a voltage of 1 V, a frequency of 300 kHz (LCR, Agilent 4980Al), and a digital display push-pull meter (SH-50, SHAHE).
