# Supplementary material for: Artificial neuromuscular fibers by multilayered coaxial integration with dynamic adaption
Source: Sci Adv. 2022 Nov 16;8(46):eabq7703. doi: 10.1126/sciadv.abq7703 (PMC9668289; doi:10.1126/sciadv.abq7703)
Supplement: Supplementary file 1 — Figs. S1 to S39 Table S1 [file sciadv.abq7703_sm.pdf]

Science Advances
AAAS

Supplementary Materials for

Artificial neuromuscular fibers by multilayered coaxial integration with dynamic adaption

Lizhong Dong *et al.*

Corresponding author: Qingwen Li, qwli2007@sinano.ac.cn; Jiangtao Di, jtdi2009@sinano.ac.cn

Sci. Adv. **8**, eabq7703 (2022)
DOI: 10.1126/sciadv.abq7703

The PDF file includes:

Figs. S1 to S39
Table S1
Legends for movies S1 to S3

Other Supplementary Material for this manuscript includes the following:

Movies S1 to S3

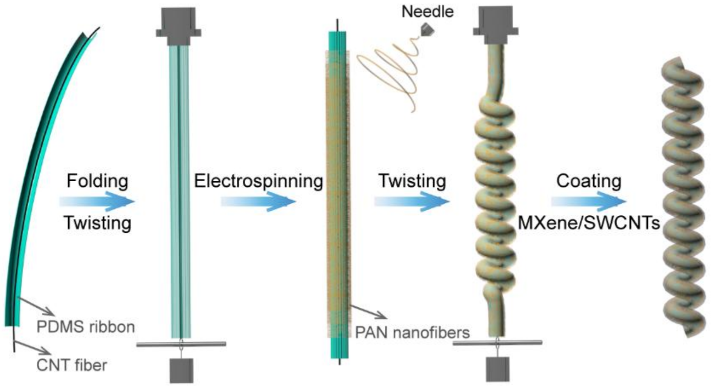

Fig. S1. Schematic illustration of the preparation of the artificial neuromuscular fibers.

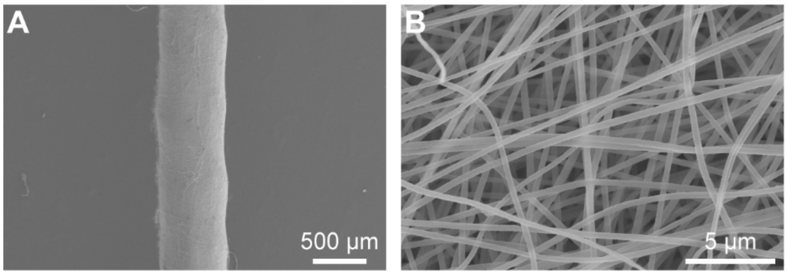

Fig. S2. SEM images of the PAN nanofibers-wrapped straight fiber.

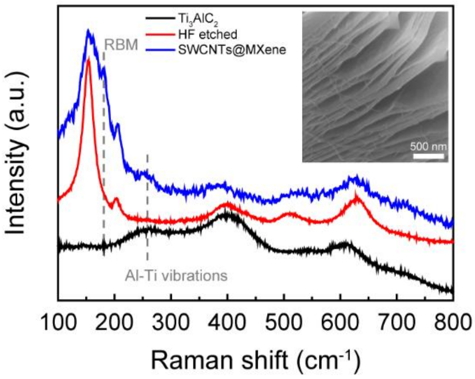

Fig. S3. Raman patterns of Ti_3AlC_2 MAX phase, freeze-dried $Ti_3C_2T_x$ MXene, and MXene intercalated by CNT. The inset is the SEM image at high magnification of the MXene/CNT sensing layer.

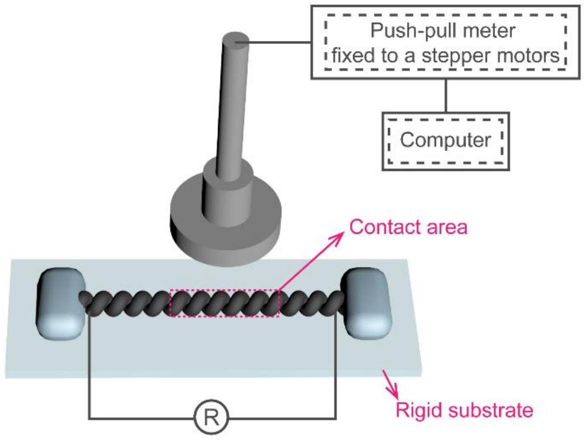

Fig. S4. Schematic illustration of the device for pressure perceptive properties testing.

The pressure perceptive properties of artificial neuromuscular fibers were tested on a rigid substrate. The ends of the fiber were fixed to a glass slide and a digital display push-pull meter was used to control the pressure applied to the fibers by a stepper motor. The initial radial length of the fiber and the axial length (rectangle with red dashed) in contact with the digital display push-pull meter were used as the basis for the calculation of the contact area. Since the initial and final contact areas of the coiled fibers under pressure are different, the space between the pitches of the coiled fibers cannot be quantitatively deducted, and uncertain deformation occurs in the horizontal direction of the coiled fibers, we have used the contact area of the coiled fibers as a fixed value during the pressure perception test to avoid the influence of uncontrollable variables as much as possible. Finally, the pressure was calculated by the formula ($P = F/S$).

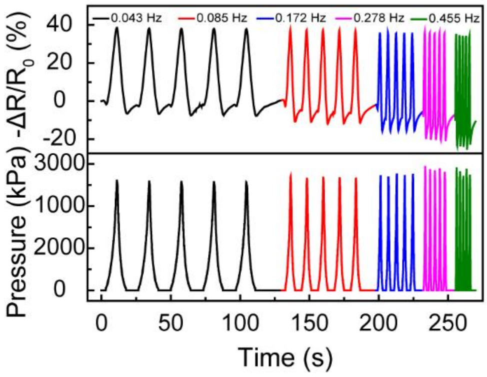

Fig. S5. The response of the relative resistance toward 0.043, 0.085, 0.172, 0.278, and 0.455 Hz frequencies under the pressure of ~2100 kPa.

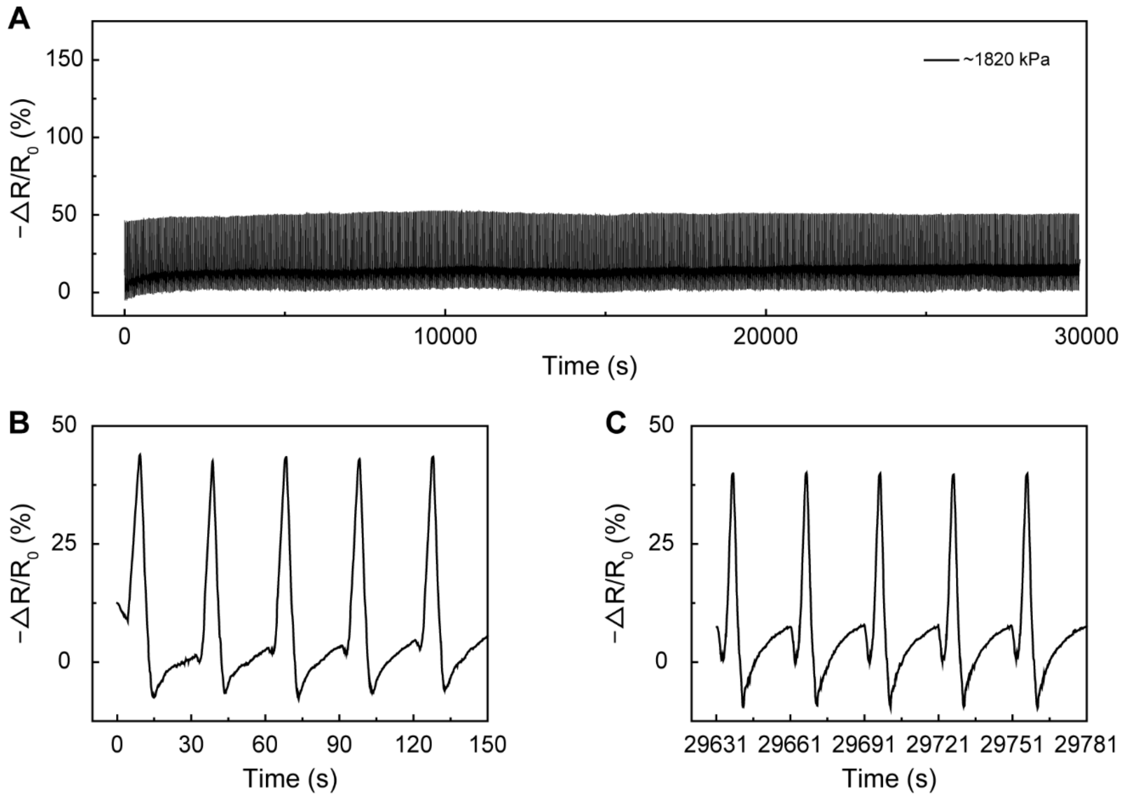

Fig. S6. Durability tested under 1000 loading/unloading cycles with the pressure and the speed of ~1820 kPa and 2.5 μm s^{-1}, respectively. (**A**) 1000 loading/unloading cycles. (**B**) The first five cycles. (**C**) The last five cycles.

The comparison of the first and the last five cycles shows that the shape and value of the peaks for the resistance signal are virtually unchanged. The artificial neuromuscular fiber was protected by the VHB tape during this process.

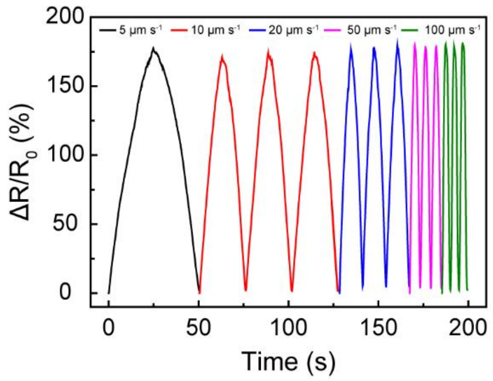

Fig. S7. The change of relative resistance as a function of strain for the artificial neuromuscular fiber under a 20% strain rate with the different stretching speeds (5, 10, 20, 50, and 100 µm s^{-1}).

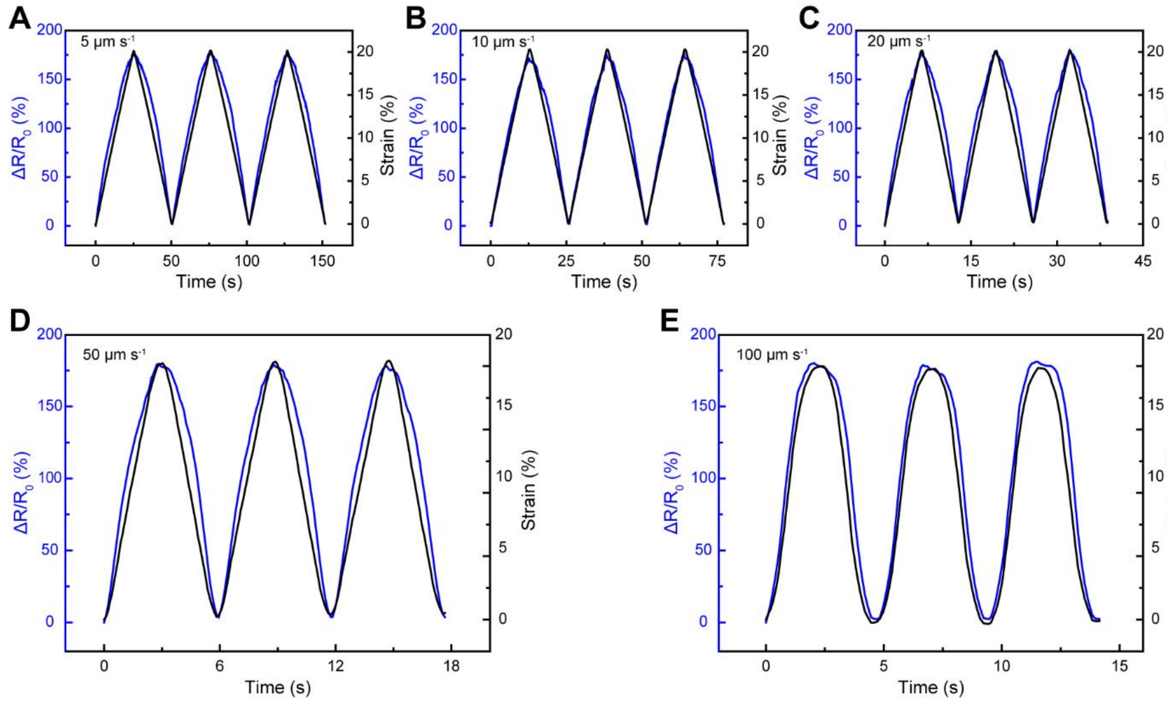

Fig. S8. The change of relative resistance as a function of time for the strain perception under a 20% strain rate with the different stretching speeds. (**A**) 5µm s^{-1}. (**B**) 10 µm s^{-1}. (**C**) 20 µm s^{-1}. (**D**) 50µm s^{-1}. (**E**) 100 µm s^{-1}.

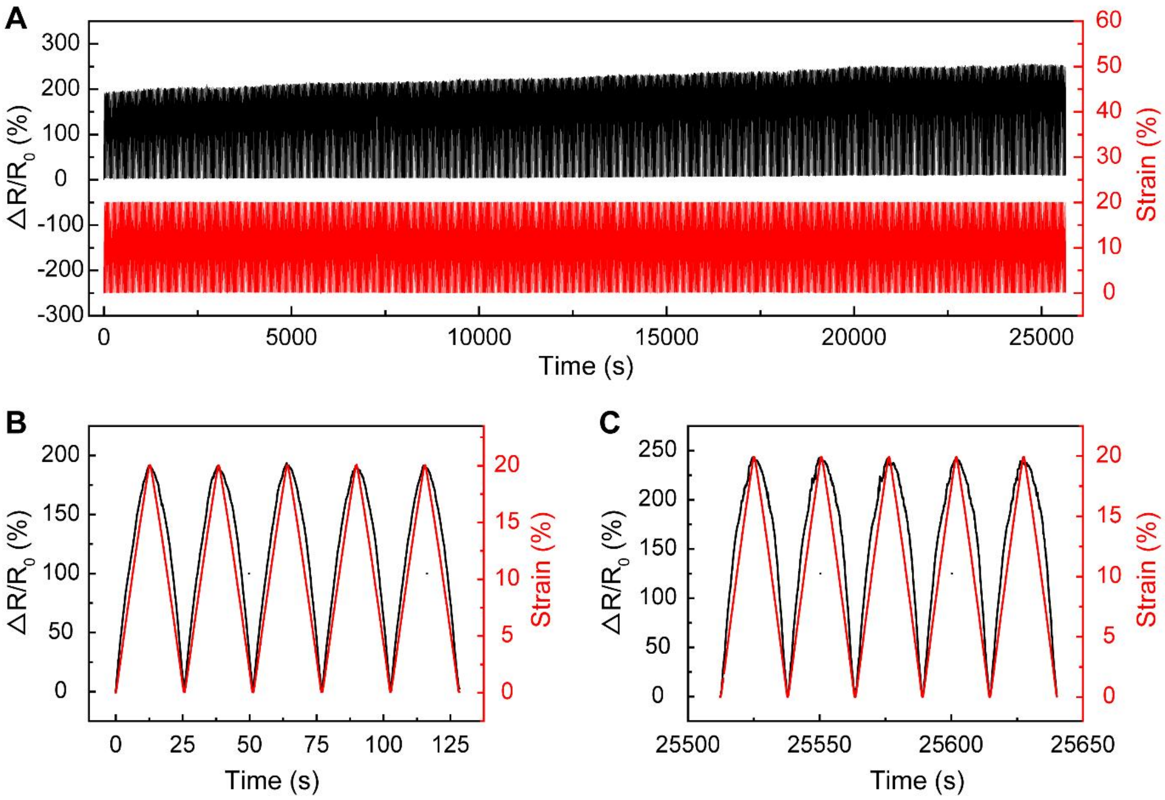

Fig. S9. Durability was tested under 1000 stretching cycles with the strain and the stretching speed of 20% and 10 µm s-1, respectively. (**A**) 1000 stretching cycles. (**B**) The first five cycles. (**C**) The last five cycles.

Comparing the first five stretching cycles with the last five stretching cycles, the change of relative resistance only experiences a slight drift.

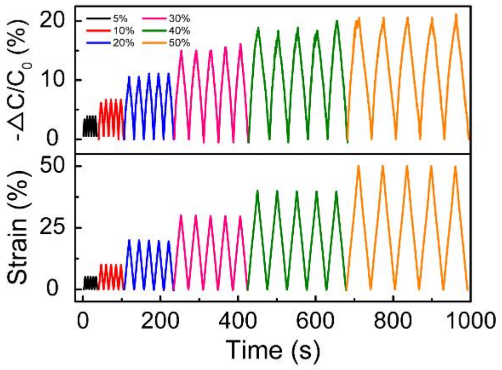

Fig. S10. The response of the relative capacitance at strains of 5%, 10%, 20%, 30%, 40%, and 50%.

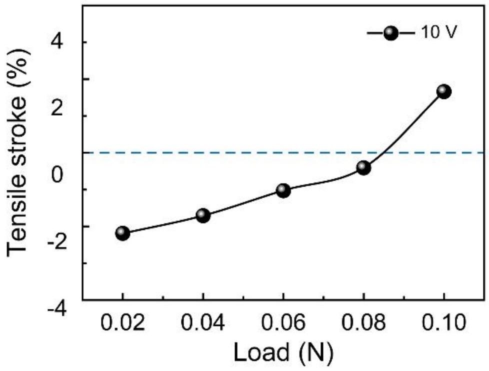

Fig. S11. The tensile actuation of the artificial neuromuscular fiber under 10 V voltage with different loads (2 g, 4 g, 6 g, 8 g, and 10 g).

The maximum elongation of the artificial neuromuscular fiber under 2 g load was 2.2%. With the load gradually increased, the elongation of the artificial neuromuscular fiber decreased to 0 and the contractile properties appeared, and the maximum contraction of the muscle fiber under 20 g load was ~20%.

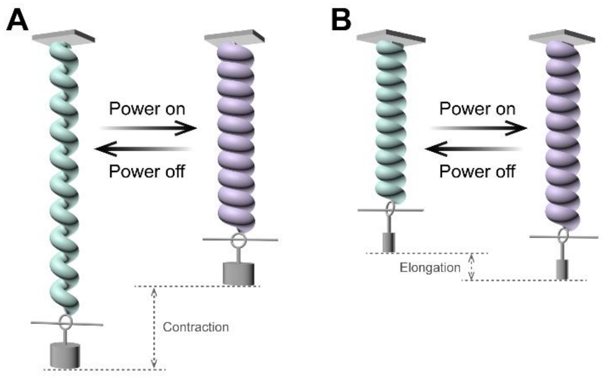

Fig. S12. Schematic illustration of the contraction mechanism (A) and elongation mechanism (B) of the artificial neuromuscular fiber.

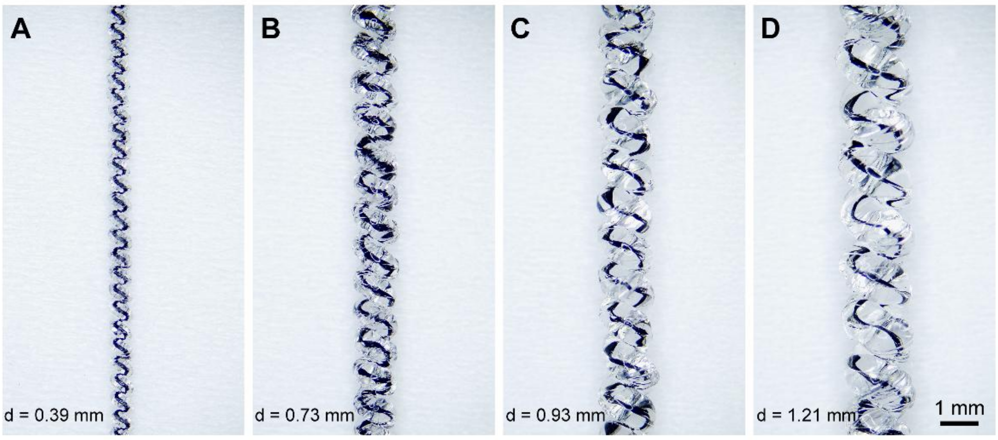

Fig. S13. Optical microscope images of the muscle fiber with different PDMS sheath thicknesses (d = 0.39, 0.73, 0.93, and 1.21 mm).

Four types of muscle fibers with different PDMS sheath thicknesses were prepared for investigating the effect of the thickness of PDMS sheath on actuation properties of the muscle fibers (the preparation parameters were shown in Table S1). To ensure that the heat generated per unit length of the CNT fiber core was the same, the maximum actuation properties of different muscle fibers under the same current of 0.14 A were tested.

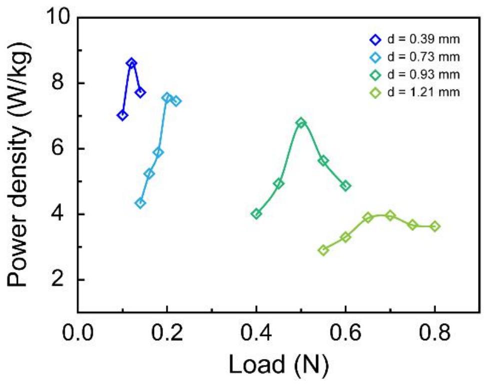

Fig. S14. The applied load dependence of corresponding power density for muscle fibers with different PDMS sheath thicknesses (d = 0.39, 0.73, 0.93, and 1.21 mm), while a current of 0.14 A was applied.

The maximum power density of the muscle fiber with a PDMS sheath thickness of 114 μm (d = 0.39 mm) under 0.14A current was 8.6 W/kg.

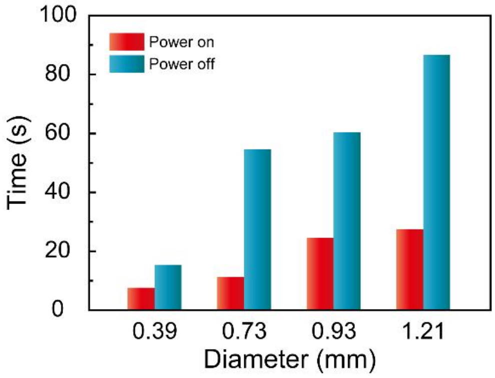

Fig. S15. The power on response time and power off recovery time of the muscle fibers with different PDMS sheath thicknesses achieving the maximum actuation.

Muscle fibers with a thin PDMS sheath thickness of 114 μm (d = 0.39 mm) could reach a maximum contraction of 10.5% when the 0.14 A current was turned on for 7 s, and could be returned to the original state when the current was turned off for 15s.

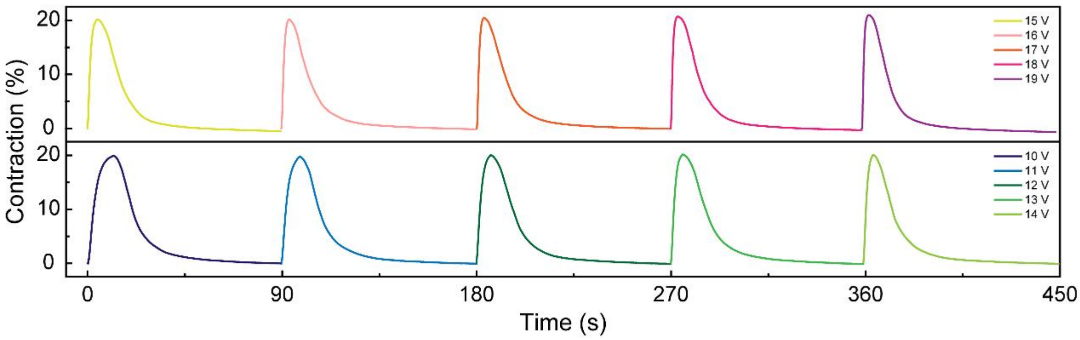

Fig. S16. The maximum contraction versus time under 20 g load at different voltages.

Although the thicker PDMS sheath improved the contraction of the muscle fibers, the responsiveness decreased significantly with the increase of the PDMS sheath thickness. The contraction response rate of the muscle fibers was improved by increasing the applied voltage without destroying the structure of the muscle fibers. The maximum contraction of the muscle fibers was maintained at ~20% as the voltage was increased from 10 V to 19 V.

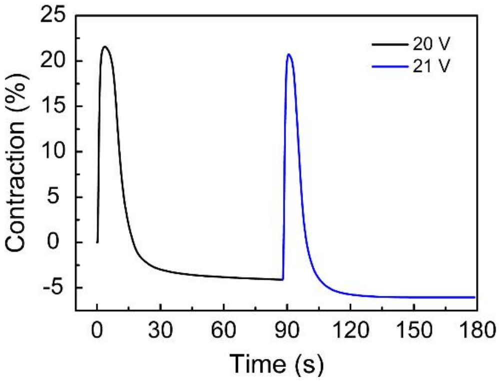

Fig. S17. The maximum contraction versus time under 20 g load at 20 V and 21V voltage.

Once the voltage exceeded 19V, the structure of the muscle fibers was destroyed after the maximum contraction was completed, thereby leading to significant creep phenomenon in the recovery process.

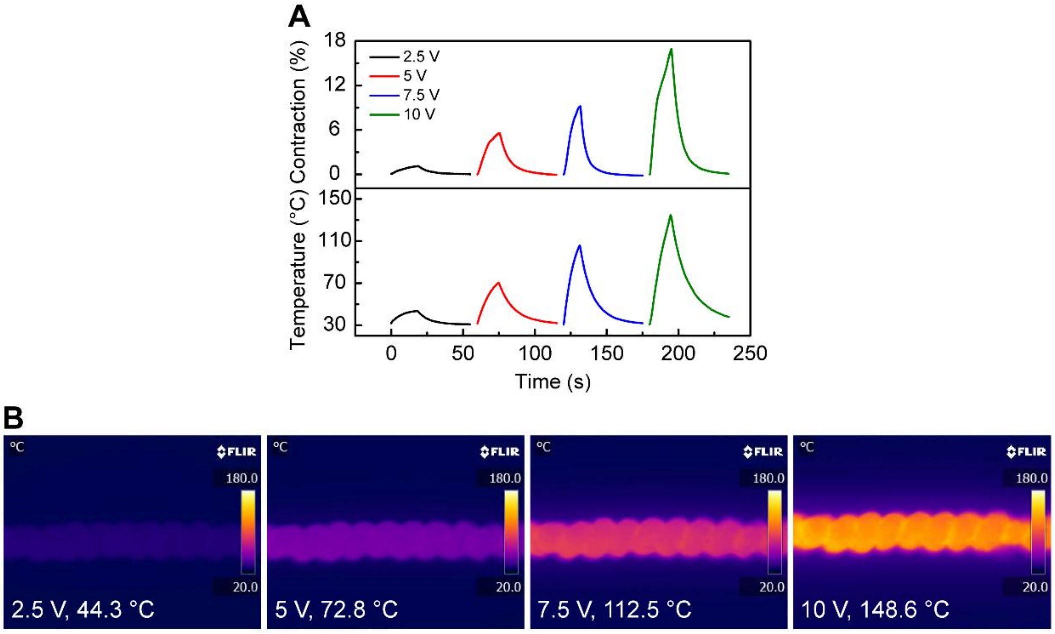

Fig. S18. The temperature change of artificial neuromuscular fiber during actuation. (**A**) The corresponding relationship between the contraction and the temperature under different voltages. (**B**) The peak operating temperatures under different voltages.

During the actuation of artificial neuromuscular fibers under different voltages, the contraction increased with the increase of temperature and showed a linear relationship with temperature. The peak operating temperatures under 2.5 V, 5 V, 7.5 V, and 10 V voltage were 44.3 °C, 72.8 °C, 112.5 °C, and 148.6 °C, respectively.

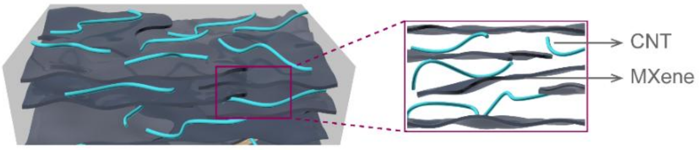

Fig. S19. Schematic illustration of the MXene/CNT 3D conductive network sensing layer.

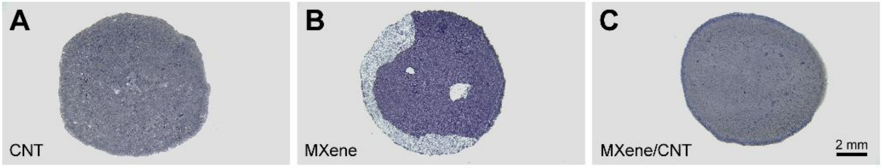

Fig. S20. Optical microscope images of the sensing layer formed by different dispersions coated on the smooth paper. (**A**) 0.1 wt% CNT. (**B**) 20 mg/ml MXene. (**C**) The MXene and CNT ratio of 5:1.

The MXene sensing layer showed poor adhesion after bending the smooth paper, resulting in an open circuit. On the contrary, the MXene/CNT sensing layer exhibited good adhesion owing to the strong interfacial interactions of the CNT network.

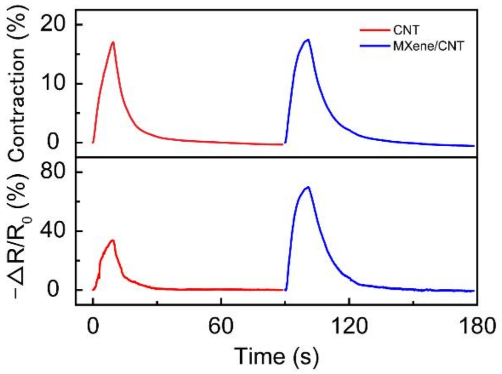

Fig. S21. The actuation and relative resistance change versus time of artificial neuromuscular fiber coated with different sensing layers.

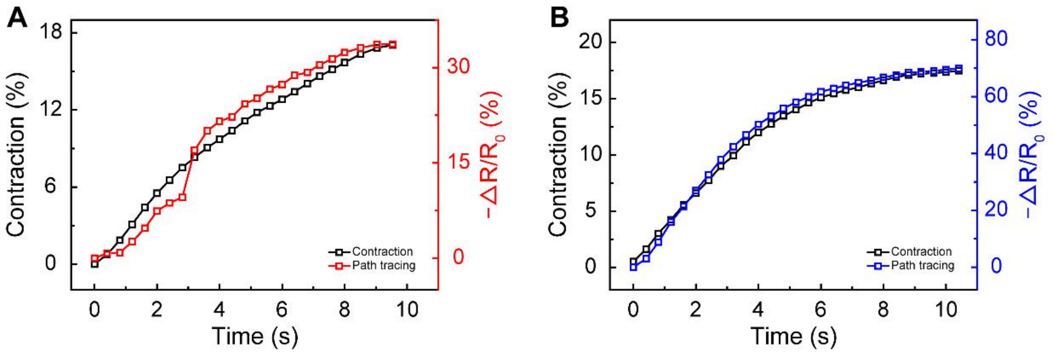

Fig. S22. The contraction and relative resistance change versus time of artificial neuromuscular fiber coated with CNT (A) and MXene/CNT (B) sensing layers under 0.14 A current.

The sensitivity of the response affected the selection of the sensing layer materials for the self-sensing muscle fibers. The artificial neuromuscular fiber coated with CNT dispersion exhibited a self-sensing function, but its signal sensitivity and linear correlation were poorer than that of MXene/CNT dispersion. This is attributed that the 3D conductive network formed by the overlapping of the MXene sheets and the CNT bundles can be very sensitive to external deformation. When the sensing layer was squeezed by external pressure or the deformation of the PDMS sheath, there was not only the contact between the bundles and bundles, but also the contact between the sheets and sheets, and between the sheets and bundles in the 3D conductive network.

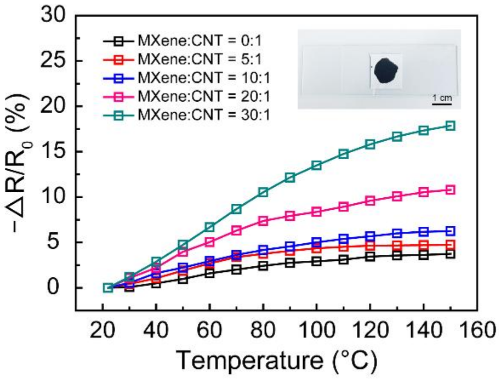

Fig. S23. The relative resistance of the sensing layers prepared by using different weight ratios of MXene and CNT with the change of temperature.

According to the different thermal resistance coefficient of MXene and CNT materials, the content of both was modulated to minimize the effect of the thermosensitive behavior for path tracing signals of the artificial neuromuscular fibers. The dispersions of different components were dropped onto smooth paper, and the relative resistance changes were measured by applying different temperatures to the dispersions after drying. The pure CNT sensing layer exhibited a very small positive thermal coefficient of resistance behavior. On the contrary, the dispersions with high MXene and CNT ratio (30:1) exhibited a large thermal coefficient of resistance behavior, which had a ~15% relative resistance change at 150 °C. After the relative content of MXene was reduced, the thermal resistance coefficient of the MXene/CNT sensing layer was correspondingly reduced. When the MXene and CNT ratio dropped to 5:1, the relative resistance change of the sensing layer at 150 °C was less than 5%, which could be negligible for the path tracing signals.

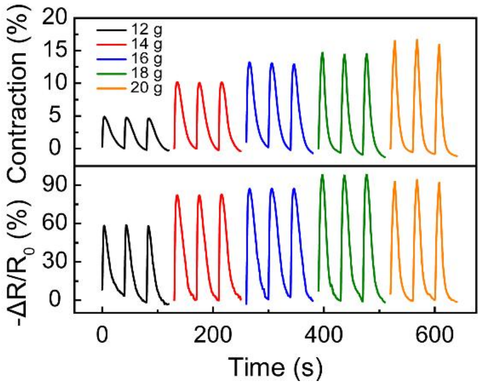

Fig. S24. Contraction and relative resistance change as a function of time at 10 V voltage under different loads (12 g, 14 g, 16 g, 18 g, and 20 g).

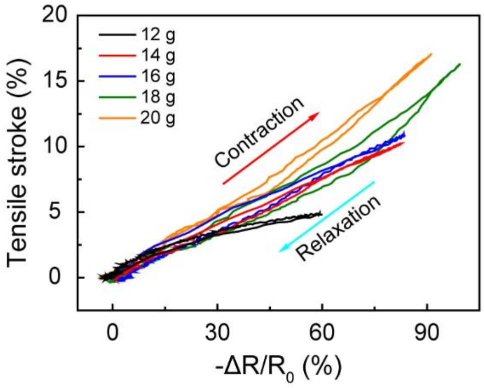

Fig. S25. The change of relative resistance as a function of the whole actuating process for the electrothermal actuation at 10 V voltage under different loads (12 g, 14 g, 16 g, 18 g, and 20 g)

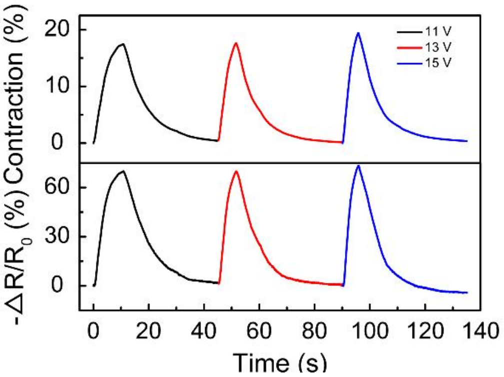

Fig. S26. Fast responsiveness of the contraction and relative resistance change for artificial neuromuscular fiber under different voltage

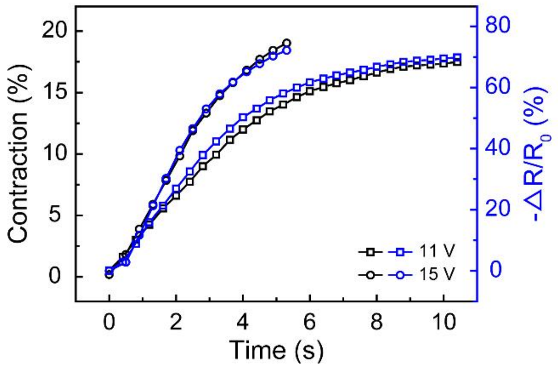

Fig. S27. The contraction and relative resistance change versus time of artificial neuromuscular fiber at different voltages.

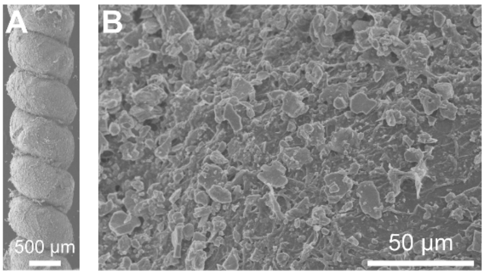

Fig. S28. SEM images of the artificial neuromuscular fiber after ~1500 actuating cycles.

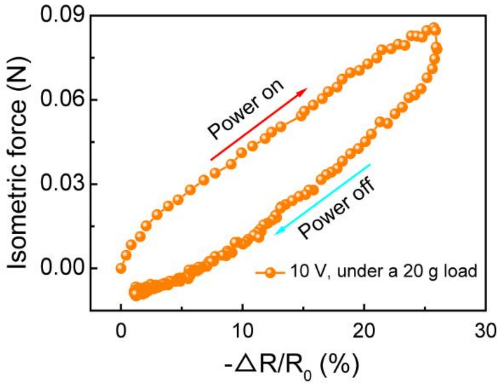

Fig. S29. The change of relative resistance as a function of the isometric force for the electrothermal actuation at 10 V voltage under 20 g load.

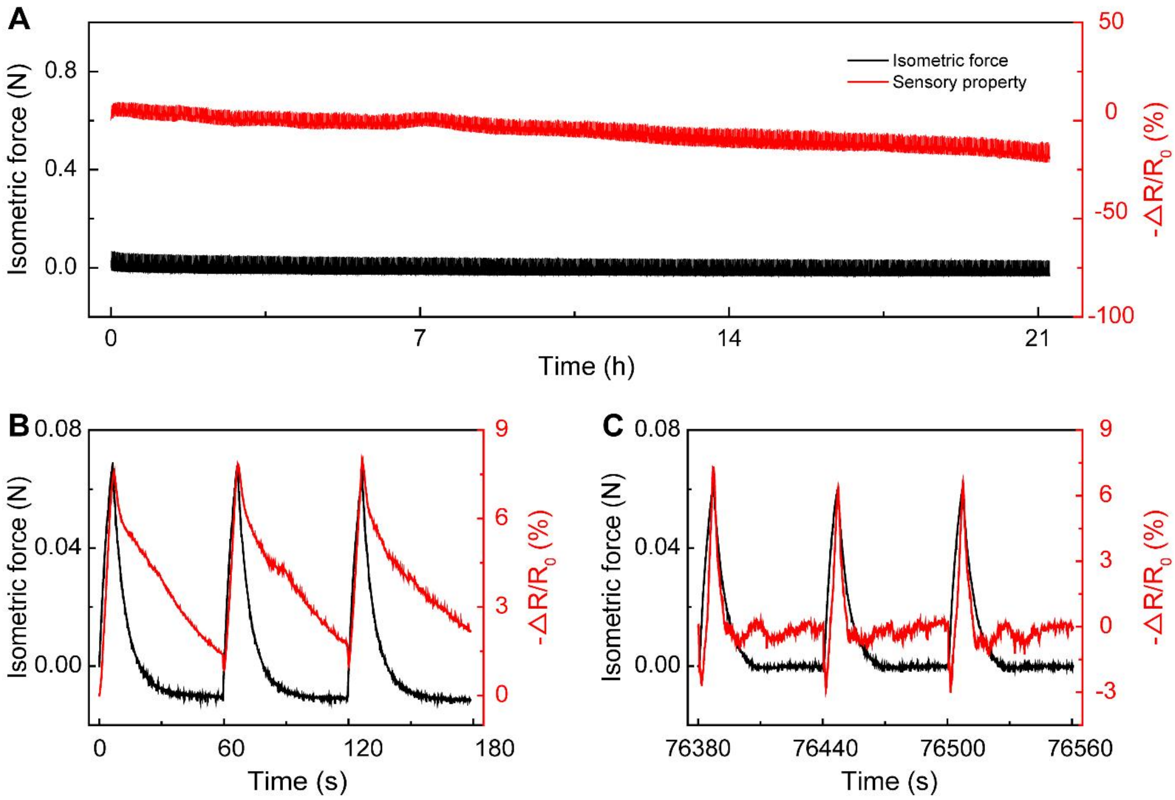

Fig. S30. Cyclic tests of the isometric force with real-time path tracing signal (under 10 V voltage with 14 g load, powering on for 8 s and powering off for 52 s) on the artificial neuromuscular fiber, 1000 actuating cycles. (**A**) 1000 actuating cycles. (**B**) The first five cycles. (**C**) The last five cycles.

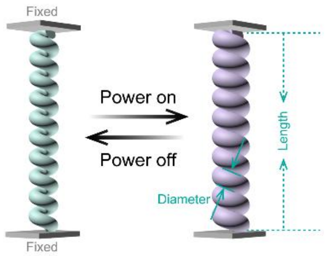

Fig. S31. The mechanism of the relative resistance of artificial neuromuscular fibers changing with isometric force.

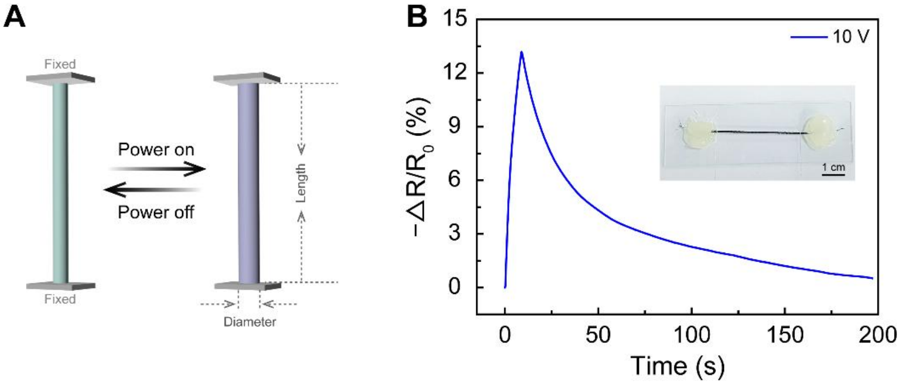

Fig. S32. The resistance change of the straight fiber fixed at both ends at 10 V voltage. (**A**) The mechanism of the relative resistance of the straight fibers fixed at both ends changing with the PDMS sheath expansion. (**B**) The relative resistance changing with the PDMS sheath expansion at 10 V voltage. The inset is the test device.

The path tracing signal of artificial neuromuscular fibers is mainly related to the microscopic deformation of the PAN substrate caused by the deformation of the PDMS sheath, which is not only affected by the change of the axial length of the coiled fibers, but also by the radial change. The resistance of the straight fibers fixed at both ends showed a decreasing trend with the expansion of the PDMS sheath, which can well explain the reason for the path tracing upon the testing process of isometric force.

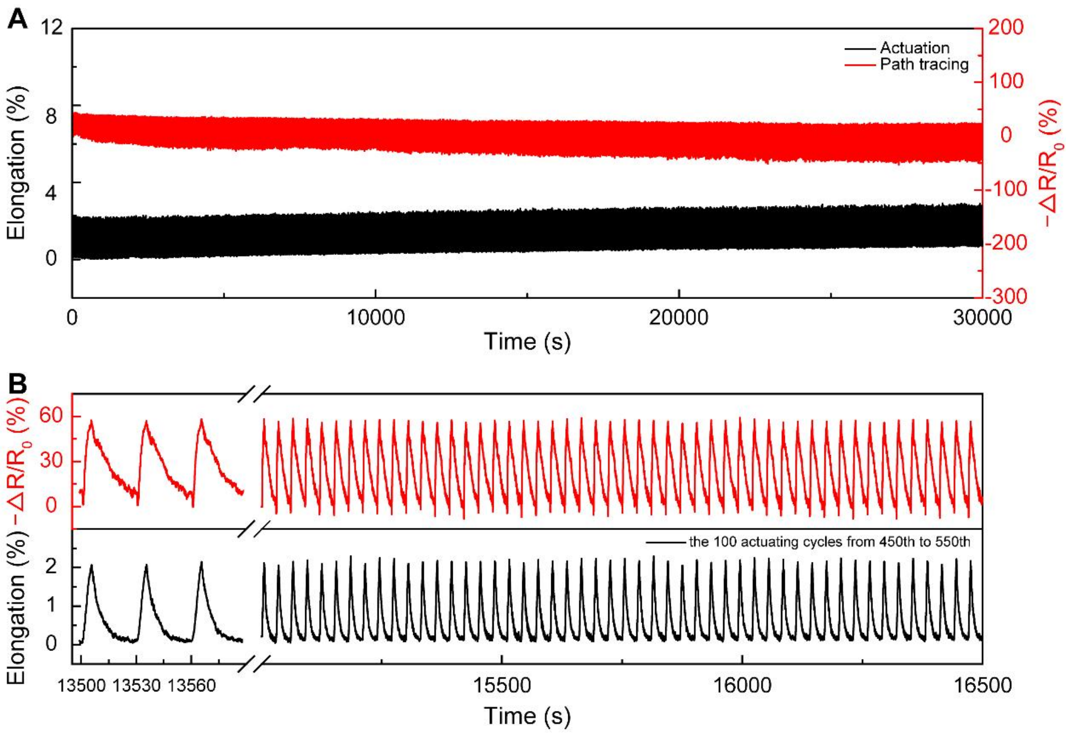

Fig. S33. Cyclic tests of the elongation with real-time path tracing signal. (**A**) Cyclic tests of the elongation with real-time path tracing (under 10 V voltage with 2 g load, powering on for 5 s and powering off for 25 s) on the artificial neuromuscular fiber, 1000 actuating cycles. (**B**) The curve of the 100 actuating cycles with real-time path tracing from 450th to 550th.

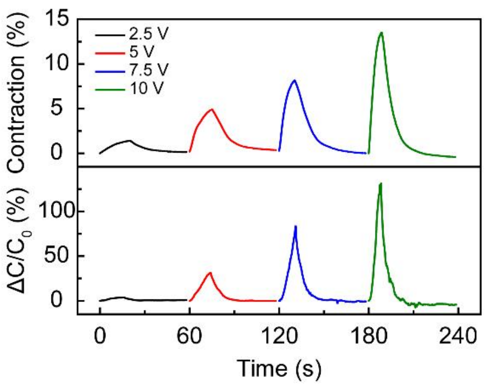

Fig. S34. Relative capacitance and contraction as a function of time under different voltages and 14 g load.

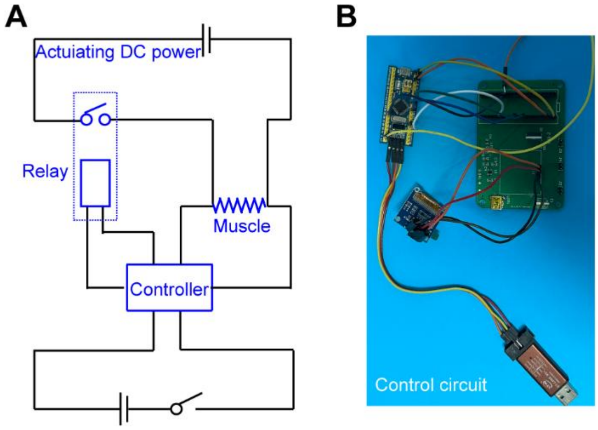

Fig. S35. Control circuit. (**A**) Schematic diagram of the electric circuit of the smart crane with perception-actuation-feedback function based on the artificial neuromuscular fiber. (**B**) Photographs of the controller and relay.

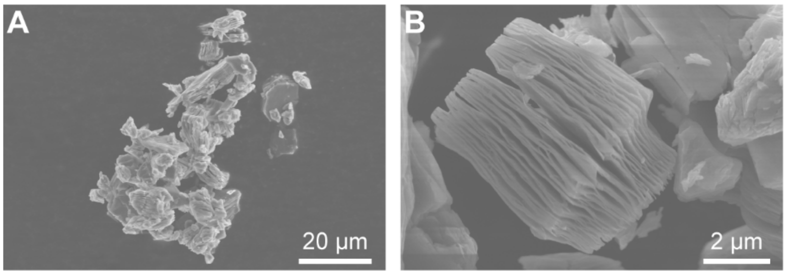

Fig. S36. Typical SEM images of the freeze-dried $Ti_3C_2T_x$ power.

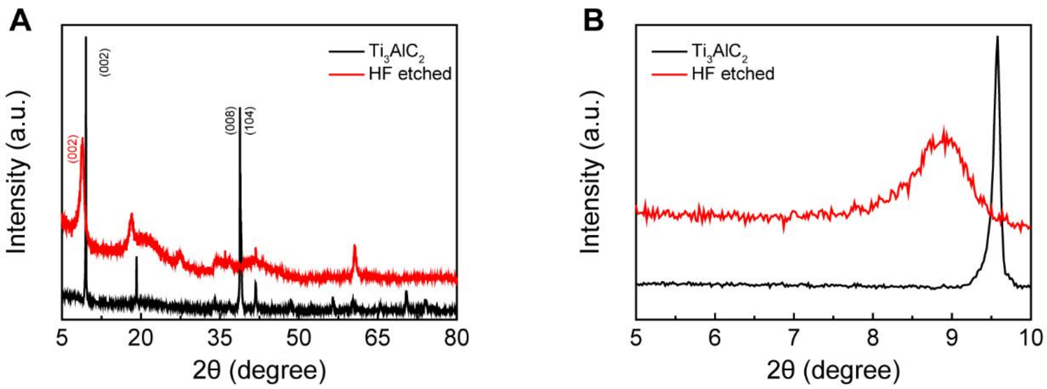

Fig. S37. XRD patterns of Ti_3AlC_2 MAX phase before and after HF treatment.

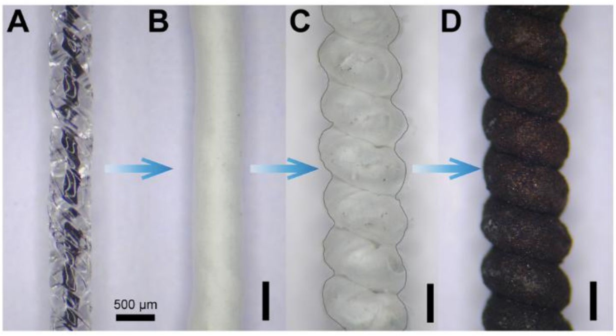

Fig. S38. The optical microscope images show the preparation of the composite fibers. (**A**) CNT@PDMS fiber (579 µm). (**B**) The PAN nanofibers-wrapped CNT@PDMS fiber (626 µm). (**C**) The PAN nanofibers-wrapped CNT@PDMS coiled fiber (626 µm). (**D**) The MXene/CNT-coated multilayer coaxial muscle fibers (646 µm).

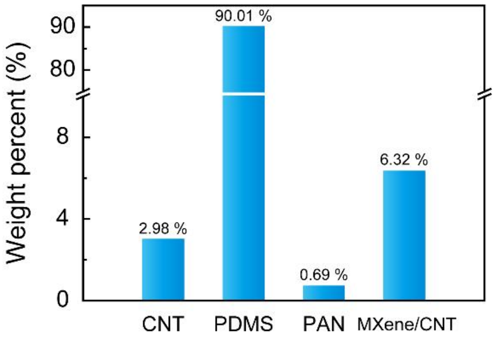

Fig. S39. The weight percent of each component of the artificial neuromuscular fibers.

The loading amount of the MXene/CNT sensing layer was not easy to quantify. The loading amount of the sensing layer was controlled by the line resistance (resistance per unit axial length of coiled fiber) of the artificial neuromuscular fibers without hanging load. Though changing the coating times of the dispersion, the line resistance of the artificial neuromuscular fibers was kept within 10-35 kΩ/cm after the sensing layer was dried. The weight percent of sensing layer in artificial neuromuscular fibers was ~6.3%.

Table S1. Preparation parameters of muscle fibers with different thicknesses of PDMS sheath.

Muscle fiber	Twisting load/g	Twist/(turns/m)	Diameter/mm	Sheath thickness/μm
1	6.5	4891	0.39	114
2	10	2421	0.73	284
2	20	1914	0.93	384
4	35	1536	1.21	524

Movie S1.
A 3D printed crane working in different modes (touchless mode by perception excitation and touch mode by piezoresistive excitation).

Movie S2.
A snail model with artificial neuromuscular fibers imitating the perception and actuation states of the tentacles of snail.

Movie S3.
A crocodile mouth capable preying on the approaching chocolate bar.
